# The Effectiveness and Feasibility of Non-Pharmacological Interventions for Reducing Behavioural and Psychosocial Symptoms of Dementia and Improving Patient Experience in Acute Care Settings: A Systematic Review

**DOI:** 10.3390/bs16050688

**Published:** 2026-04-30

**Authors:** Victoria McArthur, Susan Everington, Emily Wastell, Nmesoma Ukaji

**Affiliations:** Radiology Department, Norfolk and Norwich University Hospitals NHS Foundation Trust, Colney Lane, Norwich NR4 7UY, Norfolk, UK; susan.everington@nnuh.nhs.uk (S.E.); emily.wastell@nnuh.nhs.uk (E.W.); nmesoma.ukaji@nnuh.nhs.uk (N.U.)

**Keywords:** dementia, people with dementia, acute care, non-pharmacological interventions, behavioural and psychosocial symptoms of dementia (BPSD)

## Abstract

This review aimed to evaluate the effectiveness and feasibility of non-pharmacological interventions to reduce anxiety and agitation and improve observable wellbeing and patient engagement for people with dementia in acute hospital environments. The global increase in dementia has resulted in a substantial number of acute hospital beds occupied by people with dementia. Hospitalisation can exacerbate behavioural and psychosocial symptoms of dementia (BPSD) including anxiety and agitation, which negatively affects patient experience, safety and care. Clinical guidance recommends non-pharmacological interventions as a first-line tactic to manage BPSD. However, evidence for the effectiveness and feasibility of these interventions remains fragmented in such pressured environments. A systematic search of seven databases was conducted for studies published in the last ten years (2015–25), following the PRISMA guidelines. Fourteen studies met the eligibility criteria and included a total of 749 people with dementia. Studies used mixed interventions; music, music therapy and person-centred care highly featured and most studies reported reductions in observable BPSD during or immediately after interventions. Secondary benefits included wellbeing, reduced psychotropic medicine use, length of hospitalisation and high staff and patient acceptability. There was limited evidence for sustained effects beyond intervention. This review supports the feasibility and effectiveness of non-pharmacological interventions in acute hospitals to support dementia-inclusive, person-centred care.

## 1. Introduction

Dementia prevalence is increasing worldwide. The World Health Organization (WHO) recognises dementia as a global public health priority and it remains a growing health and social care challenge not only for the National Health Service (NHS) but for health systems internationally (WHO, 2017). An estimated 55 million people are currently living with dementia globally and this figure is predicted to rise to 150 million by 2050 (Alzheimer’s Disease International, 2022). In the United Kingdom, dementia has substantial implications for acute hospital services; the National Institute for Health and Care Excellence (NICE) estimates that at any one time approximately one in four acute hospital beds is occupied by a person with dementia (Alzheimer’s Disease International, 2022; Aranda et al., 2021). Hospitalisation for people with dementia is associated with increased risk of adverse outcomes, including higher mortality rates, particularly when admitted for an emergency or acute infection (Hapca et al., 2018; Marengoni et al., 2011; Tavares et al., 2021). Reviews of hospital outcomes have also identified increased risks of delirium, falls, hospital-acquired infections, functional deterioration, institutionalisation at discharge, all of which contribute to longer hospital stays and poorer recovery (Fogg et al., 2018). As dementia prevalence rises, demand for timely and accurate acute care, including diagnostic imaging, also increases; however, people with dementia frequently encounter barriers that negatively affect outcomes, care experience and diagnostic imaging quality.

Dementia is not as a single diagnosis or disease, but as an umbrella term describing a syndrome caused by a range of progressive neurodegenerative conditions (WHO, 2025). These conditions damage brain tissue over time, leading to cognitive impairment, behavioural changes and loss of independence in Activities of Daily Living (WHO, 2025). Globally, Alzheimer’s disease is the most common subtype, typically representing 40–60% of cases, followed by vascular dementia at around 20%, with Lewy Body dementia, Frontotemporal dementia and rarer dementias representing smaller proportions (Goodman et al., 2017; Wu et al., 2017). Although dementia subtypes differ in clinical presentation and progression, acute care environments rarely differentiate care pathways or approaches by subtype and many studies do not clearly define dementia type. For this reason and to reflect real-world clinical practice, this review includes all dementia diagnoses.

People with dementia have a significantly higher risk of hospital admission compared to those without cognitive decline (Shepherd et al., 2019) and acute hospitalisation is frequently associated with exacerbation of the behavioural and psychosocial symptoms of dementia (BPSD) which can manifest as anxiety, agitation, aggression, distress and resistance to care (Sinvani et al., 2023). These symptoms are often intensified during acute hospitalisation due to unfamiliar environments, loss of everyday routines, communication difficulties and sensory overstimulation (Butcher, 2018; Fogg et al., 2018; Johnson et al., 2011; Sinvani et al., 2023; Sommerlad et al., 2019). In older adults, even brief periods of hospitalisation or bed rest are associated with decline in mobility, independence and social engagement; for people with dementia, these declines are often faster and more severe (Fogg et al., 2018; Mok et al., 2024; O’Neil et al., 2011; Nørgaard et al., 2022). In acute settings, medical priorities may unintentionally overshadow psychosocial needs contributing to distress, disorientation and impaired communication leading to impeded care and poorer outcomes for this group of people (Butcher, 2018).

Acute hospital environments present distinct challenges compared with long-term or residential care homes. Time-pressures, staffing constraints, and high-stimulus environments coupled with limited dementia-specific training can hinder the delivery of person-centred care (Ballard et al., 2018; Brossard Saxell et al., 2021). In such contexts, BPSD can interfere with clinical assessments, treatment delivery and diagnostic procedures including imaging, leading to delayed care, increased length of stay and poorer patient experience and outcomes (Fogg et al., 2018; Sinvani et al., 2023; Johnson et al., 2011). Despite the growing recognition of the need for dementia-friendly hospitals, there remains a gap between policy recommendation and routine practice in acute care.

Historically pharmacological interventions have been commonly used to manage anxiety and BPSD in hospital settings (Kales et al., 2015). However, there is an increasing awareness that acute care needs more dementia-friendly environments. Clinical recommendations from NICE advocate non-pharmacological treatments as the first-line approach for managing distress and agitation in people with dementia (NICE, 2019). Similarly, the WHO’s Global Action Plan on Dementia emphasises dignity, autonomy and person-centred care advocating for dementia-inclusive health systems that adapt care environments and practices to cognitive impairment (Reich et al., 2022; WHO, 2017). Despite these recommendations, evidence supporting the implementation of non-pharmacological interventions in acute hospital settings remains fragmented, with much of the existing data focused on long-term or community environments.

Emerging evidence suggests that sensory-based, creative and psychosocial interventions, may reduce anxiety and agitation in people with dementia (Edmans et al., 2021; McArthur et al., 2024; Karrer et al., 2023; NICE, 2019; Reich et al., 2022). Music and music therapy (MT) has been shown to influence physiological stress responses, including reductions in heart rate, blood pressure and cortisol levels while also enhancing mood and social engagement (Finn & Fancourt, 2018; Hole et al., 2015). Person-centred care approaches and multi-component interventions that consider identity, sensory needs and preserved abilities have shown promise in reducing BPSD and improving care and experience (Cooper et al., 2012; Douglas et al., 2004). However, the effectiveness, feasibility and acceptability of these interventions within the constraints of acute care remain unclear.

To address this gap, this systematic review aims to synthesise the evidence of effectiveness and feasibility of non-pharmacological interventions for reducing the BPSD and improving patient experience in acute hospital settings. The primary objective is to evaluate the impact of these interventions on BPSD. BPSD encompasses a range of non-cognitive symptoms, including agitation, anxiety, mood disturbance and behavioural dysregulation. In this review, outcomes relating to agitation, anxiety, and emotional distress were interpreted within the broader construct of BPSD. Secondary objectives include assessing the feasibility of deliverability and acceptance by patients, staff and caregivers as well as noting other outcomes such as wellbeing, pain, staff care practices, length of hospital stay. This review seeks to inform clinical practice and support the integration of evidence-based, person-centred interventions in high-pressured acute hospital settings, including diagnostic imaging pathways.

## 2. Materials and Methods

The Preferred Reporting Items for Systematic Reviews and Meta-Analyses (PRISMA) guidelines and recommendations (PRISMA, 2020) were followed when designing and conducting this systematic review (PROSPERO registration number CRD42024618782). The PRISMA checklist for systematic reviews was followed (Supplementary Materials).

### 2.1. Eligibility Criteria

The Population, Intervention, Comparator, Outcome and Study (PICOS) framework established the criteria for inclusion before conducting the literature search. Studies had to be in the English language. These terms were developed with an experienced systematic review librarian.

#### 2.1.1. Population

Eligible studies included people with dementia who had been diagnosed with any kind of dementia. Studies of patients with delirium or acute confusion were excluded. Patients had to be in an acute hospital setting (including acute geriatric hospitals).

#### 2.1.2. Intervention

The eligible studies used any non-pharmacological intervention used to lower a broad range of manifestations of BPSD including anxiety and other behavioural and/or neuropsychiatric symptoms. These BPSD could be measured in any manner, whether a validated anxiety-specific one or observational.

#### 2.1.3. Comparator

Studies with any control or no control and other kinds of intervention were included.

### 2.2. Outcomes of Interest

#### 2.2.1. Primary Outcome

Studies were included if they measured any BPSD with an intervention. Levels could be reported as any response or a self-reporting measure such as State Trait Anxiety Inventory (STAI).

#### 2.2.2. Secondary Outcomes

- Wellbeing or other quality-of-life reports
- Reduction in pain
- Staff knowledge and care management
- Length of stay
- Feasibility of delivery
- Acceptance of intervention

### 2.3. Study Design

Experimental comparative studies of any non-pharmacological intervention during a visit or procedure in an acute hospital. These were not restricted to randomised controlled study designs as the authors were aware that there may be a limited number of such trials.

### 2.4. Exclusions

- Systematic reviews
- Pharmacological intervention
- Care homes
- Not published in English language
- Not published between 2015 and 2025

### 2.5. Information Sources and Literature Search Strategy

A structured literature search of seven databases (Medline, EMBASE, EMCARE, AMED, CINAHL, PycINFO, Cochrane (including CENTRAL)) and was conducted on 03/2025 for relevant studies (a repeat search was done in December 2025). A ten-year date limit was applied to all publications. A librarian was consulted on the keywords mapped with the PICO framework (Appendix A.1). Only full text articles in English were included. Additional citation searching was also used examining relevant systematic reviews and included publications.

### 2.6. Selection Process

Retrieved articles were exported to Excel and duplicates removed by the first author. Titles and abstracts were screened independently by at least two co-authors to assess for eligibility. Discrepancies were discussed and resolved until consensus by another co-author. Full text screening was completed by two authors supported with a third to resolve disagreements. All co-authors participated in this stage.

### 2.7. Data Extraction

Data were extracted from the included studies based on a standardised data extraction form (Cochrane Public Health Group Template, 2024) slightly modified and developed a priori. Data were extracted by one author and reviewed by all other authors. Feasibility and acceptance of interventions were marked if considered in the studies.

### 2.8. Data Items

- Data extracted included the following:
- Author, date, country
- Description of study design
- Setting and sample size
- Type of intervention
- Intervention details
- Outcomes measured
- Results
- Main findings
- Feasibility of intervention and acceptance was considered
- Study quality

### 2.9. Quality Assessment

Studies are presented according to the hierarchy of evidence outlined by York Guidance (Khan et al., 2001), with RCTs reported first, followed by designs with a greater risk of bias. Study quality was assessed using the Mixed Method Appraisal Tool 2018 (MMAT, 2018) (Appendix A.2); this was chosen to reflect the heterogeneity of the included studies, generating a rating out of five stars. Methodological quality was appraised independently by at least two co-authors using the Mixed Method Appraisal Tool (MMAT), with consensus reached through discussion. Although most studies met several quality criteria and were therefore considered of moderate to high methodological quality, common limitations included small sample sizes, non-randomised or observational designs, and short follow-up periods, which may increase the risk of bias and should be considered when interpreting the findings.

### 2.10. Synthesis

Given the substantial heterogeneity in study populations, methodologies, anxiety measures and outcomes, a meta-synthesis was not appropriate. A narrative synthesis was undertaken, with key findings tabulated and followed established guidance (Popay et al., 2006). This approach enabled meaningful comparison across overlapping, though not directly comparable, domains. It also allowed integration of quantitative and qualitative evidence, providing a richer understanding of how interventions operate within acute healthcare settings and how the environments respond to the needs of complex patient groups such as people with dementia. Key findings were extracted by one author and verified by another co-author. They were grouped by type of non-pharmacological intervention, measures used and key findings. These were further synthesised to generate a conceptual pathway.

## 3. Results

### 3.1. Study Selection

**Figure 1:**
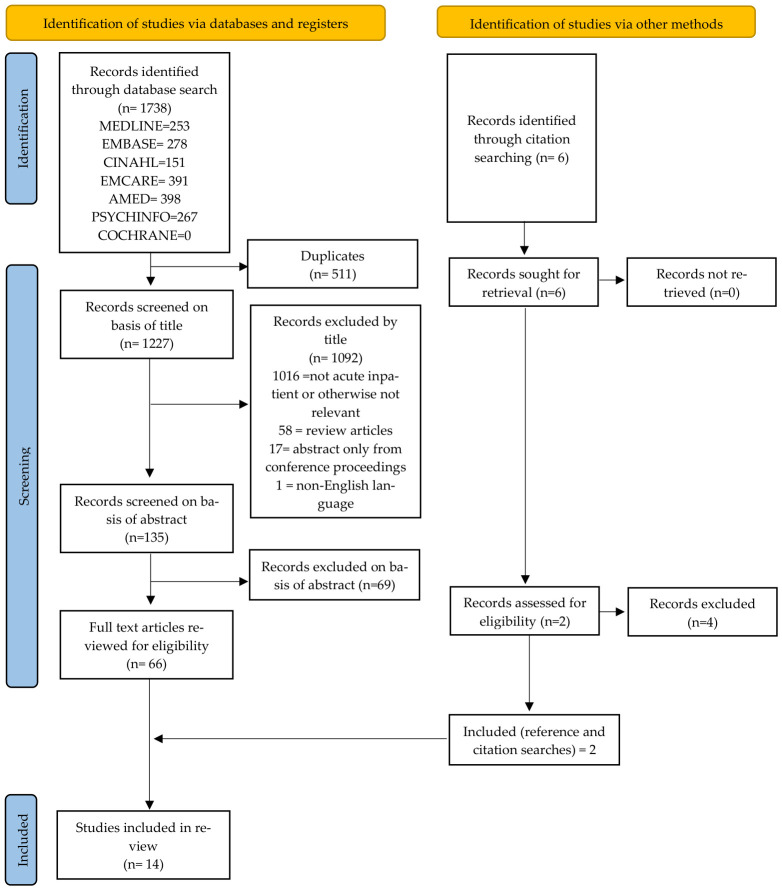
PRISMA Flow diagram for included articles.

A summary of the study selection is shown in Figure 1. This illustrates the results of the selection process. Of the 135 studies identified, 66 were screened by full text for review and 14 were eligible for inclusion in this review.

### 3.2. Study Characteristics

The included studies are summarised in Table 1.

A total of 749 patients and an unknown number of staff participated in 14 studies. Sample size varied from 13 to 175 people with dementia. There was a mix of study methodologies, including RCT, mixed method, pre- and post-intervention and observational studies. The studies were carried out in nine countries.

Three studies used an RCT design, the remaining studies used a mix of quasi-experimental, feasibility, pilot, pre-/post-test and mixed method designs. The majority of the studies were carried out in Europe (n = 6) with three completed in the US. The interventions were delivered by both professional therapists and hospital staff.

### 3.3. Summary of Interventions Used to Lower Anxiety and Agitation

#### Primary Outcome

The non-pharmacological interventions can be divided into the following categories:

1. Music-Based Interventions, including: MT, CMT, Individualised music listening, live/recorded music, tailored music sessions and music delivered online (Abeywickrama et al., 2025; Álvarez Gómez et al., 2019; Belenchia, 2023; Daykin et al., 2018; Dimitriou et al., 2022; Cheong et al., 2016; Lee et al., 2023; Mandzuk et al., 2018; Munsterman et al., 2024; Pitkänen et al., 2019; Thompson et al., 2023).
2. Multi-Component Psychosocial Interventions, such as: person-centred care (PCC), mixed therapies such as validation therapy, aromatherapy/massage, and individualised activity programmes around identity, sensory abilities, emotional needs and preserved skills (Chenoweth et al., 2022; Dimitriou et al., 2022).
3. Sensory and Support-Based Interventions: for example, PARO robot therapy or enhanced staffing models to offer support beyond standard dementia care (Dasgupta et al., 2021; Munsterman et al., 2024; Sinvani et al., 2023).

Both primary and secondary outcomes are summarised in Table 2. A variety of structured and unstructured observations, pre- and post-intervention observation periods, focus groups with carers, and interviews were used to understand experience and impact.

### 3.4. Risk of Bias in the Studies and Quality of the Evidence

This was examined using MMAT and is presented in Table 1. The ratings can be found in Supplementary Materials (Table S1). The table is presented according to the study design. All of the studies included varied from high to medium quality as assessed independently by at least two authors.

### 3.5. Overall Pattern

Most studies reported reductions in observable agitation during or immediately after non-pharmacological interventions, particularly with personalised or familiar music, PCC approaches and multi-component therapies. However, evidence of sustained or consistent reductions in anxiety or agitation across sessions was variable. Anxiety as a physiological, subjective state or self-reported construct was less directly measured, but behavioural indicators showed meaningful reductions.

### 3.6. Results of the Synthesis

**Figure 2:**
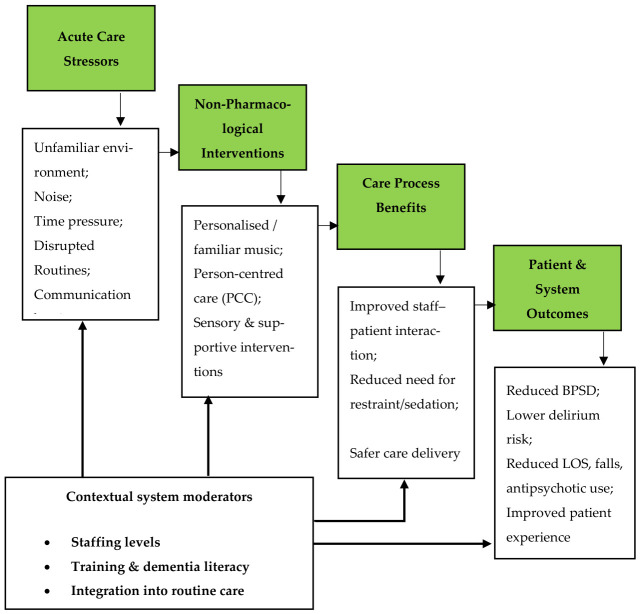
Conceptual pathway for acute care of people with dementia.

The flow chart (Figure 2) represents a conceptual framework proposed by the authors in which acute care stressors prompt the implementation of non-pharmacological interventions, leading to improvements in care processes that subsequently influence patient and system-level outcomes. System pressures act as moderating factors, shaping the strength and direction of these relationships across the pathway.

## 4. Discussion

This systematic review synthesised evidence from 14 studies involving 749 people with dementia, examining non-pharmacological interventions to reduce BPSD in acute hospital settings. Overall, the findings indicate that non-pharmacological approaches, particularly music-based interventions and person-centred care can reduce observable agitation and distress in people with dementia during acute care encounters. These interventions were consistently reported as feasible and acceptable to patients and staff and align with current dementia guidance.

### 4.1. Effectiveness of Music-Based Interventions

Music-based interventions emerged as the most frequently studied and consistently effective approach for reducing BPSD in acute care. Across multiple designs, including RCTs, quasi-experimental and observational studies, personalised or familiar music was associated with reductions in agitation (varying measures used PAS, CMAI, NPI-Q), fewer disruptive incidents, including improvements in mood, engagement and emotional wellbeing.

Importantly, in-person MT was more effective than virtual delivery or no intervention, suggesting that the relational and sensory components of live music are critical in acute care contexts. The findings strongly align with NICE dementia guidance (NG97) and the World Health Organization’s Global Action Plan on the Public Health Response to Dementia, both of which emphasise person-centred, non-pharmacological approaches as first-line strategies for managing distress and agitation in people living with dementia (NICE, 2018; WHO, 2017).

While behavioural and neuropsychiatric symptoms were rarely measured directly using physiological or self-report measures, behavioural indicators commonly associated with anxiety, such as agitation, aggression, restlessness and distress, were reduced. In acute care contexts where cognitive impairment limits reliable reporting both from staff and people with dementia, these behavioural proxies should be seen as meaningful. However, the varied or lack of direct anxiety measurements represents an important evidence gap.

It should be noted that the effects of music were often time-limited, with several studies reporting that benefits did not persist beyond the intervention period (Mandzuk et al., 2018; Pitkänen et al., 2019). This aligns with existing literature suggesting that music functions as a regulator of distress rather than a disease-modifying intervention (de Witte et al., 2020). Nonetheless, even short-term reductions in agitation are clinically important in acute care, where immediate cooperation, safety, and procedural success are priorities, particularly relevant for settings such as emergency care and diagnostic imaging (Smith et al., 2025).

### 4.2. Role of Person-Centred and Multi-Component Interventions

PCC-based interventions demonstrated significant reductions in agitation and delirium, alongside improvements in care quality. However, these effects were less consistently sustained among patients with longer hospital stays, greater cognitive impairment, and exposure to unstable ward environments. This highlights that PCC is most effective when embedded within supportive systems that include trained staff, consistent routines and organisational commitment (Moore et al., 2017). Multi-component interventions combining music, validation therapy, sensory approaches or enhanced staffing also showed promise, particularly in reducing irritability and caregiver distress, though isolating the contribution of individual components remains challenging.

### 4.3. Secondary Outcomes and System-Level Benefits

Beyond lowering BPSD, non-pharmacological interventions were associated with a range of secondary benefits relevant to patient safety and system performance. Reduction in falls, length of stay and lower psychotropic medication use were observed in intervention groups alongside improved staff engagement and general wellbeing. These findings are particularly important given the well-documented risks associated with prolonged hospitalisation and antipsychotic use in people with dementia.

In particular, in-person delivery of MT was associated with significantly fewer disruptive incidents compared with online or absent interventions, highlighting the importance of human presence and relational engagement. Sensory and support-based interventions, including robotic pet therapy also showed promise in reducing delirium and medication use, suggesting potential adjunctive roles where staffing resources are limited.

### 4.4. Feasibility, Acceptability and Sustainability

A key strength of this review is its focus on feasibility and acceptability within acute care. Most interventions were reported as feasible to deliver alongside routine clinical care, with minimal adverse events and high levels of patient engagement and acceptance. Staff consistently described interventions as satisfying to deliver and reported enhanced understanding of patients’ needs and personhood.

However, evidence for sustained effects beyond intervention periods was mixed. Several studies noted that reductions in agitation did not persist once interventions ceased, particularly in patients with longer hospital stays or advanced cognitive impairment. This finding likely reflects systemic constraints rather than intervention ineffectiveness and underscores the importance of embedding non-pharmacological strategies into routine care rather than delivering them as isolated or time-limited activities.

### 4.5. Limitations of the Evidence Base

Despite encouraging findings, the evidence base remains limited by small sample sizes, underpowered studies, lack of blinding and heterogeneity in outcome measures. Anxiety as one measure of BPSD was rarely measured using validated specific measures or physiological tools (e.g., STAI), with most studies relying on behavioural proxies. This limits conclusions about BPSD and specifically anxiety as a distinct construct and underscores the need for more standardised outcome frameworks. Additionally, few studies examined longer term outcomes or implementation beyond pilot phases.

Dementia severity was rarely reported on and could influence the success of interventions. This lack of consistent reporting of dementia severity across studies represents an important limitation, as agitation and behavioural symptoms may present differently across stages of dementia (and types of dementia) and may influence responsiveness to non-pharmacological interventions.

Blinding is rare in such studies and the heterogenous nature of study designs (with few RCTs) limits confidence in generalising; however, there is consistency in the positive direction of the intervention effect, if not the magnitude. A mix of study settings, even though all acute, has added complexity with differing staffing, routines and patient acuity. All acute settings, however, are all experienced by people with dementia as urgent, unexpected hospital care and the two most commonly used interventions; music-based and PCC, are adaptable to time-limited, high-throughput settings. In fact, the dominance of music, emerging as the strongest theme, is perhaps because of its immediacy, feasibility and low cost, suiting such environments. This is supported by staff-based findings which showed such interventions were accepted and feasible and aligned with acute care goals such as comfort, cooperation and reduced distress during care episodes. Acute care implementation depends heavily on staff capacity, training, and organisational culture which was not examined in detail in this review.

A range of outcome measures was used across the included studies because agitation and behavioural disturbance in dementia are multidimensional constructs encompassing behavioural, emotional, and engagement-related components. Consequently, studies frequently employ different validated instruments such as agitation scales, neuropsychiatric symptom inventories and observational emotion or engagement measures to capture these related but distinct domains. This range of outcome measures limits the ability to directly compare interventions, but also highlights the need for standardised scales.

### 4.6. Implications for Acute Care and Future Research

Although the strength of evidence varied across intervention categories and study designs, a small number of randomised or controlled studies reported statistically significant reductions in agitation or behavioural symptoms, while several quasi-experimental and observational studies primarily demonstrated short-term behavioural improvements during intervention periods. Differences in intervention intensity and duration may also have influenced outcomes, with more frequent or personalised music sessions generally associated with stronger behavioural responses. In addition, the wide range of outcome measures used to assess BPSD limits direct comparison of effect sizes across studies and contributes to variability in reported findings. Taken together, these factors suggest that although the overall direction of evidence supports the potential value of non-pharmacological approaches in acute care, the strength and consistency of effects remain variable.

Despite these limitations the findings indicate that non-pharmacological interventions, especially personalised music and PCC, are generally feasible and acceptable for use during acute care encounters and may reduce observable distress and agitation in people with dementia. However, the evidence for sustained effectiveness remains limited; several studies reported that benefits did not persist beyond the intervention periods. This indicates that current evidence for non-pharmacological interventions in acute care remains preliminary and should be interpreted with caution.

Nevertheless as emergency admissions for people with dementia rise steeply (Torjesen, 2020), interventions that are low cost, minimal risk and adaptable are particularly suitable for transfer into high-throughput environments such as emergency care and diagnostic imaging. Future research should prioritise adequately powered trials in acute hospital settings, incorporate standardised measures of BPSD, report on degree of dementia, investigate the system moderators that act as barriers and evaluate implementation strategies that support sustainability across care pathways.

## 5. Conclusions

This systematic review demonstrates that non-pharmacological interventions, particularly personalised music-based approaches and person-centred care, can reduce BPSD, such as anxiety and agitation for people with dementia in acute hospital settings. Although effects are often time-limited, even short-term reductions in BPSD are clinically significant in high-pressure environments where cooperation, safety, and timely care are critical. The findings align strongly with NICE and WHO guidance advocating non-pharmacological strategies as first-line interventions and highlight additional system-level benefits, including reduced reliance on psychotropic medications and improved staff confidence. Importantly, this review extends existing guidance by highlighting the feasibility of such interventions in highly pressured environments, such as acute care, which are characterised by rapid turnover, competing priorities, and limited staffing.

## Figures and Tables

**Table 1:** Characteristics of the included studies. A variety of outcome measures were used, these are listed at the end of the table.

First Author, Year, Country	Study Design	Setting & Sample Size	Study Design and Analysis Approach	Type of Intervention and Details	Outcomes Measured	Key Findings	Key Conclusions	Feasibility	Study Quality Grading
Lee et al. (2023) Australia	Single centre two-arm randomised control feasibility trial.	Geriatric management and evaluation unit in metropolitan hospital n = 21	Participants randomly allocated to intervention or control group	Playlist of personally curated music, Control group: routine medical care. Participants listened—1 h for 5 days (3–4 pm). Time chosen because of ‘sundowning effect’	Feasibility assessed with 45 min interviews with delivery team. Patients PAS was recorded and CGI completed pre-/post-trial.	Intervention feasible and satisfying to deliver. Challenged use of psychotropic medications and increased staff engagement, PAS data higher in intervention group.	Feasible to deliver music intervention alongside clinical care. Assisted with engagement and increased efficiency in some clinical tasks. PAS result inconclusive possibly due to being underpowered.	Yes	4 ****
Dimitriou et al. (2022) Greece	Cross-over randomised control trial	Neurological Departments of Thessaloniki and Athens n = 60	Six non-pharmacological interventions including music	Assigned into six mixed therapy groups composed of: Validation Therapy, Aromatherapy and massage, Music Therapy (MT) (preferred music). For five days.	Patients and caregivers assessed for baseline measures of: MMSE, ACE, GDS, FRSSD, NPI. And after interventions.	Interventions can reduce irritability in people with dementia and lower caregiver distress. Significant finding in a combination of therapies.	MT combined with other therapies may reduce agitation.	Not clear	3 ***
Munsterman et al. (2024) US	Unblinded, randomised controlled trial	Hospital, acute n = 38	15 min with Robotic pet therapy or 15 with normal human visitor	PARO-‘Personal robot’ in Japanese, a socially assistive robot. Compared to empathetic human visitor. Three visits (15 min/day), capped if visible agitation. Concluded on day six, or earlier if discharged	MoCA and CAM. Secondary outcomes—use of restraints and medicines	17 human visitor participants and 21 PARO. Participants spent > time in human group vs robot. PARO group significantly less medication and delirium. Use of restraints had no difference.	PARO robotic pet has promise to reduce delirium use and psychotropic medications in acute care. Challenges in acute care are limited staffing, there remains the possibility of training volunteers in robots.	Maybe	4 ****
Belenchia (2023) US	Quasi-experimental, paired samples, pre-post design	Acute care hospital n = 21	Individualised music listening over ten-week period (measurements taken before and after)	Individualised music intervention to reduce agitation in people with dementia. (Individualised music identified by interviewing patients, family members or caregivers on music preferences downloaded onto iPods.)	PAS scored pre- and post-intervention individualised music listening on (five point Likert Scale (0–4, 0 = no reaction, 4 = striking caregiver) in four behaviours groups	Average PAS post-test lower than pre—reduced agitation in all four behavioural groups. Greatest in motor agitation, then aberrant vocalisation, resisting care and the smallest change for aggressiveness.	Individualised music is an effective intervention reducing agitation in people with dementia. Staff responded positively and would consider a treatment in managing BPSD. Patients responsive to personalization of music, effects were immediate.	Yes	3 ***
Dasgupta et al. (2021) Canada	Pre-post case-series methodological study.	General medicine sub-acute unit. n = 13 (11 documented people with dementia)	Control-no., pre and post intervention measures for case studies.	Individualised interventions incorporating: (1) sense of identity; (2) sensory abilities; and (3) enhanced understanding of current needs. Two-week period, outcomes prior and post.	Primary outcomes: Participants engagement; Need for assistance and mobility support. Secondary Outcomes: behaviour, restraint use, calls to security, falls and neuroleptic use.	Unique interventions with a PCC approach-considering participant prior to cognitive impairment in a very dynamic with different care providers interacting with the patient. Some evidence for less neuroleptic use.	Post-intervention, most participants had reduced agitated behaviours. Non-pharmacologic interventions feasible in acute care. Low implementation due to lack of engagement. Optimise by including family members, increased staff education and technology.	Yes	3 ***
Sinvani et al. (2023) US	Prospective non-randomised pilot feasibility trial.	Two tertiary hospitals. n= 158	One dementia unit staffed with nurse assistant (NA) plus Patient Engagement Specialists (PES) and another staffed with routine care (NA).	Extra PES compared to standard care (NA with normal dementia care and education training). Effectiveness of an added layer of staff (PES) working 8 h shifts compared to normal care performed by NA	NPI-Q scores, plus use of restraints, psychoactive medications, Length of stay (LOS) and falls. Satisfaction obtained using family caregiver.	Although a difference in NPI-Q scores did not differ significantly, overall rated good/excellent.	Demonstrated feasibility of using NA and training for hospitalised people with dementia. No difference between the NA and NA + PES—due to baseline differences in control/intervention, and variability of dementia.	Yes	3 ***
Abeywickrama et al. (2025) UK	Single-arm intervention trial	Two inpatient wards for people with advanced dementia n = 17	Before and after intervention measures.	Weekly group music (Ward 1). Patients free to join/leave. Ward 2 one-to-one sessions due to needs of patient. Tailored music was dynamic, responding to people with dementia. Some sessions used instruments (maracas). 5 weeks.	Before and after measures of NPI-Q and MiDAS used at the beginning (first five minutes and during intervention) (most significant five minutes)	NPI-Q scores before and after. For Delusion, Motor Disturbances, and Agitation scores significantly reduced post-intervention. Significant improvements in: Interest, Response, and Enjoyment of MiDAS items	Positive delivery of music interventions to people with dementia. A useful, cost-effective, non-pharmacological intervention, alongside routine care. Multi-component interventions decrease BPSD and improve mood, cognition and communication. Staff observations positive.	Yes	4 ****
Chenoweth et al. (2022) Australia	Non-randomised two group (control and treatment) pre- post-intervention study	Acute ward and mixed surgical ward on tertiary hospital n = 47	Control group -staff trained on dementia and delirium care, Intervention group -staff trained in PCC (person-centred care).	PCC- comprising four parts: baseline audit, PCC training, VIPS framework. Three timepoints collected: baseline; day 4–5 (T2) and for subgroup T3 (more than 8 days after baseline).	Outcomes measured CMAI QUIS Secondary outcomes CAM, ADL Iatrogenic harms including falls and injuries, psychotropic medicines use and LOS	CMAI lower in PCC group and QUIS higher. Secondary outcomes-risk of CAM decreased at T1. Falls and injury higher in control group at all times. More discharges to aged-care homes in control.	PCC in acute hospital reduced BPSD and delirium at T2 compared to usual care. Not sustained with longer stays. PCC reduces BPSD when supported by system (staffing, stable routines, and organisation). Greater cognitive impairment/comorbidities/ busy wards contributed to unsustained results.	Yes, if supported by staff	4 ****
Pitkänen et al. (2019) Finland	Observation-interventional (Benchmark controlled trial	Acute psychogeriatric ward (n = 175)	Intervention group (n = 86) singing and listening to music. Control (n = 89) no intervention.	Weekly singing, live, recorded music, and dancing	NPI, MMSE. BI and ADCS-ADL	No evidence that music interventions reduced anxiety, agitation or aggression, and no overwhelming benefit of physical exercise.	No significant differences between groups. Possible reduction in anxiety and improved sleep in the intervention group.	N/A	5 *****
Mandzuk et al. (2018) Canada	Quality improvement, pre-/post-intervention, observational.	Acute care facility. (n = 20)	Pre, during and post-intervention behaviours observed.	Personalised playlist created on portable music device with aid of carers, staff and individual. Listening sessions lasted 30 min on 10 sessions.	Effectiveness of personalised music as distraction from boredom/distress, participation in reminiscence, and greater engagement.	9/10 adults engaged in positive, calm appreciative responses including reminiscence. These responses were limited and not lasting.	Personalised music engaged people with dementia in positive actions and distraction. There was no comparison to non-individualised music. Music effects did not last post the sessions. Staff felt there were benefits.	Yes	4 ****
Álvarez Gómez et al. (2019) Spain	Cross-sectional observational	Acute geriatric ward (n = 32)	Recorded music	15 min of music (ranging from more to less relaxing) played over headphones and patients responses observed.	Observed responses (three healthcare providers), generalised responses were recorded before and after.	27/32 patients showed positive responses (smiling, sang, danced, exhibited pain relief), two indifferent, one aggressive. Familiar melodies of youth liked.	Music stimulus, appropriate used and timely help relax and divert people with dementia.	Yes	3 ***
Cheong et al. (2016) Singapore	Within subject design (participant acting as own controls) pre–post-intervention	Acute Care setting Geriatric Hospital (n = 25)	Pre-post intervention observations, control = no interventions. 5 min interval observations. Analysed for mean difference in engagement and effect.	All subjects (groups or one-to-one) 30 m observation with no intervention Day 1 (1/2 h ×3 period). Followed by 30 m of Creative Music Therapy (CMT)-music improvisation; playing familiar songs and listening	MPES Lawton OERS	Positive MPES (higher during CMT, and MPES lower. Positive LOERS (pleasure general alertness) higher during CMT, and negative OERS (anger, anxiety, sadness) lower.	CMT effected mood and engagement, could have a supplementary role to support treatments. With known resistance to care and nursing procedures in people with dementia, CMT in daily care could improve compliance and cooperation.	Yes	4 ****
Daykin et al. (2018) UK	Exploratory sequential mixed methods	Acute elderly care hospital (n = 85)	Weekly inclusive participatory music activity	Unstructured observation of music sessions plus brief interviews post-music. Interviews with focus group of carers	Quantitative data on falls, length of stay, antipsychotic prescriptions and ArtsObs to record mood, distraction, relaxation and happiness.	Decrease in LOS, falls, reduction in antipsychotic medication during music. Observational—music has positive effect in clinical environment.	Overall strong positive effect on clinical environment. Responses to music are elicited wide range of emotions.	Yes	4 ****
Thompson et al. (2023) UK	Mixed methods retrospective observational	Two inpatient psychiatric wards for people with dementia (n = 37)	Retrospective gathering of distress behaviour incidents (Datix) correlated to when MT sessions were scheduled.	MT sessions (usual practice), 2× weekly. Until COVID-19, sessions were delivered online to Ward 1 and it was not possible to arrange for Ward 2 due to very vulnerable patient group.	Disruptive or aggressive behaviour occurred every 7.1% of days with in-person therapy, compared with 32.1% and 30.6% of days for online and no therapy, respectively.	Number of incidents on in-person MT days were significantly lower than number of incidents on days with online MT.	In-person MT showed reduction in reported rates of disruptive and aggressive behaviour compared to none. Other outcomes- staff perceptions: MT was a helpful for people with dementia, could lift mood, calm agitation and possibly reduce distress	Yes	4 ****

Alphabetical list of abbreviations used. ACE—Addenbrooks Cognitive Exam; ADL—Activities of Daily Living; ArtsObs—psychometric instrument designed to measure changes resulting from art-based interventions; CAM—Confusion assessment method to measure delirium acute change, inattention, disorganised thinking, altered consciousness; CGI—Clinical Global Impression assesses patients clinical condition before intervention and global improvement; CMAI—Cohen-Mansfield Agitation Inventory a 29 rating questionnaire of behavioural/neuropsychiatric symptoms that can occur in dementia; FRSSD—Functional Rating Scale for Symptoms of Dementia; GDS—Global Deterioration Scale; LOERS—Lawton Observed Emotion Rating Scale to measure affect with five types of emotion recorded included: pleasure, general alertness, anger, anxiety/fear, and sadness; MiDAS—Music in Dementia Assessment Scales which can be transformed into five VAS; MMSE—Mini Mental State Exam; MoCA—measures: attention, concentration, executive function, visual spatial skills, language; MPES—Menorah Park Engagement Scale developed to assess levels of engagement in adult day care patients during Montessori-based dementia programming; NPI-Q—Neuropsychiatric Inventory Questionnaire; PAS—Pittsburg Agitation Scale. Four types of behaviour associated with agitation: aberrant vocalisation, motor agitation, aggressiveness, and resisting care. The tool uses a 5-point Likert scale ranging from 0 to 4. A rating of 0 indicates the behaviour is not present; PCC—person-centred care; QUIS—Quality of Interactions Schedule an observational measure of care quality; VAS—(Visual Analogue Scale) items (Levels of Interest, Response, Initiation, Involvement and Enjoyment; VIPS = V = Valuing and respecting the people with dementia, I = treating the people with dementia as an sentient Individual, P = seeing the service care from the Perspective of the people with dementia, S = creating a positive Social environment for care services.

**Table 2:** Key findings of primary and secondary outcomes.

Outcome Category	Measures Used	Key Findings
**Primary outcome**		
BPSD	MPES, OERS, CMAI, PAS, NPI-Q	Significant reductions in agitation during CMT, higher constructive/passive engagement and lower self-engagement/non-engagement (Cheong et al., 2016). Reduced negative emotions (anger, anxiety, sadness) and increased pleasure/alertness (Cheong et al., 2016; Thompson et al., 2023). Scores significantly lower post-intervention, especially motor agitation and vocalisation (Belenchia, 2023; Dimitriou et al., 2022). Agitation, motor disturbance and delusions improved post-music (Belenchia, 2023; Chenoweth et al., 2022; Dasgupta et al., 2021; Dimitriou et al., 2022; Sinvani et al., 2023). Patients showed calm, positive responses; familiar music strongest effect (Abeywickrama et al., 2025; Álvarez Gómez et al., 2019; Mandzuk et al., 2018). In-person MT days had significantly fewer disruptive/aggressive incidents than online or no therapy (Belenchia, 2023; Thompson et al., 2023). PCC group: CMAI significantly lower than controls (Chenoweth et al., 2022). Some studies reported no lasting or consistent reduction outside session periods (Dasgupta et al., 2021; Pitkänen et al., 2019)
**Secondary Outcomes**		
Wellbeing/Quality of Life	OERS (pleasure, alertness), MiDAS (interest, response, enjoyment), ArtsObs (mood), qualitative staff/patient reports	Marked improvements in pleasure, interest, alertness, responsiveness and enjoyment (Cheong et al., 2016; Daykin et al., 2018; Álvarez Gómez et al., 2019; Mandzuk et al., 2018; Thompson et al., 2023). Observations showed reduced boredom/distress and greater social engagement (Cheong et al., 2016; Mandzuk et al., 2018). Human interaction preferred, but PARO also improved engagement and reduced delirium (Munsterman et al., 2024).
Pain Reduction	Observed behavioural responses (e.g., smiling, dancing)	Some participants displayed observable pain relief behaviours during music sessions (more relaxed posture, smiling, decreased distress) (Álvarez Gómez et al., 2019). However, they were not consistently measured quantitatively.
Staff Knowledge & Care Management	Feasibility interviews; QUIS (care quality); qualitative feedback	Interventions increased staff engagement, improved understanding of personhood, and challenged psychotropic use (Lee et al., 2023; Sinvani et al., 2023). PCC group showed significantly higher care quality (Dasgupta et al., 2021). Staff reported delivery was feasible and satisfying (Abeywickrama et al., 2025; Belenchia, 2023; Chenoweth et al., 2022; Dasgupta et al., 2021; Lee et al., 2023; Mandzuk et al., 2018; Sinvani et al., 2023).
Length of Stay (LOS)	Hospital LOS data; discharge destination	Decrease in LOS during music activity periods (Daykin et al., 2018). Lower risk of delirium and more stable behaviour in intervention groups contributed to reduced LOS (Sinvani et al., 2023). Control group had significantly more discharges to aged-care facilities (Chenoweth et al., 2022).
Feasibility of Delivery	Staff interviews and session observations	Delivery consistently found feasible across settings, including personalised music playlists, PCC interventions and group activities (Dasgupta et al., 2021; Lee et al., 2023; Mandzuk et al., 2018; Sinvani et al., 2023). Dynamic, personalised music selection well tolerated.
Intervention acceptance	Patient observed responses; caregiver surveys (e.g., 10-item FCG); participation rates	High acceptance, positive engagement, positive or pain-relieved behaviours; very few adverse responses (Álvarez Gómez et al., 2019; Mandzuk et al., 2018). Family caregiver satisfaction rated good–excellent (Dimitriou et al., 2022). Both PARO and human visitor groups well accepted; human preferred socially, PARO associated with reduced medication use (Munsterman et al., 2024).
Additional Clinical Outcomes	Falls, restraint use, psychotropic medicines, CAM, antipsychotic prescribing	Falls and injuries lower in intervention groups (Daykin et al., 2018). Reduced psychotropic/neuroleptic use (Dasgupta et al., 2021; Daykin et al., 2018). Delirium risk significantly decreased post-intervention (Chenoweth et al., 2022; Munsterman et al., 2024). PARO group had less medication use and less delirium than human visitor group (Munsterman et al., 2024). Music and PCC interventions linked with lower antipsychotic use and improved broader safety indicators (Daykin et al., 2018).

## Data Availability

No new data were created or analysed in this study. Data sharing is not applicable to this article.

## Supplementary Materials

The following supporting information can be downloaded at: https://www.mdpi.com/article/10.3390/bs16050688/s1, Table S1: PRISMA CHECKLIST.

## Abbreviations

The following abbreviations are used in this manuscript:

BPSD	Behavioural and Psychosocial Symptoms of Dementia
WHO	World Health Organisation
NICE	National Institute for Health and Care Excellence
NHS	National Health Service
MT	Music Therapy
CMT	Creative Music Therapy

## Appendix A

### Appendix A.1. Example of Search Terms Used

exp *Exercise/

“life-story book*”.mp.

*Odorants/ or scent*.mp.

*Smell/

*Music Therapy/ or *Music/

songs.mp.

“playlist*”.mp.

“cognitive stimulation”.mp.

“environmental stimulation”.mp.

*Caregivers/

*Family/

*Touch/ or *Therapeutic Touch/

comforter*.mp.

blanket*.mp.

“fiddle blanket*”.mp.

talking.mp.

“therapy activity”.mp.

“therapy activity kit*”.mp.

“non-pharmacological”.mp.

1 or 2 or 3 or 4 or 5 or 6 or 7 or 8 or 9 or 10 or 11 or 12 or 13 or 14 or 15 or 16 or 17 or 18 or 19 exp *Dementia/

20 and 21

exp *Hospitalization/ or “Acute Care”.mp.

22 and 23

limit 24 to english language

### Appendix A.2. Table MMAT Outcomes for All Papers

**Table:**

**Authors**		**Confirm Acute Setting**	**1. Screening Questions (for All Types)**		**2. RCTs**					**Rating**
**2. RCTs**			**S1. Are there clear research questions?**	**S2. Do the collected data address the research questions?**	**2.1. Is randomization appropriately performed?**	**2.2. Are the groups comparable at baseline?**	**2.3. Are there complete outcome data?**	**2.4. Are outcome assessors blinded to the intervention provided?**	**2.5 Did the participants adhere to the assigned intervention?**	
Lee et al. (2023)	Single centre two-arm RC feasibility trial	Y	Y	Y	Y	Y	Y	N	Y	4 ****
Dimitriou et al. (2022)	Cross -over RCT	Y	Y	Y	Y	Y	Y	N	Y	3 ***
Munsterman et al. (2024)		Y	Y	Y	Y	Y	Y	N	Y	4 ****
**3. Quasi-experimental (same appraisal as RCT, see above)**										
Belenchia (2023)	Quasi-experimental	Y	Y	Y	N/A (paired)	N	Y	N	Y	3 ***
Dasgupta et al. (2021)	Pre-post quasi-experimental	Y	Y	Y	N/A (paired)	N	Y	N	Y	3 ***
Sinvani et al. (2023)	Non-RCT pilot feasibility	Y	N	Y	N	N	Y	N	Y	3 ***
Abeywickrama et al. (2025)	Before and after time series	Y	Y	Y	N/A (paired)	Y	Y	N	Y	4 ****
**4. Non-RCT**					**4.1. Are the participants representative of the target population?**	**4.2. Are measurements appropriate regarding both the outcome and intervention (or exposure)?**	**4.3. Are there complete outcome data?**	**4.4. Are the confounders accounted for in the design and analysis?**	**4.5. During the study period, is the intervention administered (or exposure occurred) as intended?**	
Chenoweth et al. (2022)	Non-RCT two group	Y	Y	Y	Y	Y	Y	Y	Y	4 ****
**5. Observational (same appraisal as Non-RCT)**										
Pitkänen et al. (2019)	Non-RCT two group	Y	Y	Y	Y	Y	Y	Y	Y	5 *****
Mandzuk et al. (2018)	Prospective, Controlled Non-RCT	Y	Y	Y	Y	Y	Y	Y	Y	4 ****
Álvarez Gómez et al. (2019)	Non-RCT two group	Y	Y	Y	Y	?	?	N	Y	3 ***
Cheong et al. (2016) Singapore	Prospective, Controlled Non-RCT	Y	Y	Y	Y	Y	Y	N	Y	4 ****
**6. Mixed methods**					**6.1. Is there an adequate rationale for using a mixed methods design to address the research question?**	**6.2. Are the different components of the study effectively integrated to answer the research question?**	**6.3. Are the outputs of the integration of qualitative and quantitative components adequately interpreted?**	**6.4. Are divergences and inconsistencies between quantitative and qualitative results adequately addressed?**	**6.5. Do the different components of the study adhere to the quality criteria of each tradition of the methods involved?**	
Thompson et al. (2023)	Mixed methods retrospective	Y	Y	Y	N	Y	Y	Y	Y	4 ****
Daykin et al. (2018)	Exploratory sequential mixed methods	Y	Y	Y	N	Y	Y	Y	Y	4 ****

Papers were rated out of 5 ***** by all authors based on their MMAT outcomes. Where 5 ***** was felt to be the highest quality paper. Consensus was sought through discussion.
